# Fibrocartilaginous embolism: a rare cause of cervical spine infarction

**DOI:** 10.1097/MS9.0000000000002005

**Published:** 2024-04-04

**Authors:** Widad Abu Mayyala, Nawras Abu Aqeel, Afnan W.M. Jobran, Farah Shaheen, Mohammed Abdulrazzak, Yousef Alhroub

**Affiliations:** a Faculty of medicine, Palestine Polytechnic University, Hebron, Palestine; b Faculty of Medicine, Al Quds University, Jerusalem, Palestine; c Faculty of Medicine, University of Aleppo, Aleppo, Syria; d Neurosurgery department, Hebron Governmental Hospital, Hebron, Palestine

**Keywords:** case report, fibrocartilaginous embolism, ischaemic myelopathy, quadriplegic, spinal cord

## Abstract

**Introduction and importance:**

One of the uncommon causes of ischaemic myelopathy is fibrocartilaginous embolisation, which results from the intersomatic disc nucleus pulposus becoming embolised into the spinal vasculature during Valsalva-like manoeuvres.

**Case presentation:**

A 29-year-old female patient presented to the authors’ emergency department with general weakness, dizziness, and an inability to move her right hand after a minor trauma. These symptoms deteriorated suddenly until the patient became quadriplegic. The clinical picture and MRI led to a diagnosis of fibrocartilaginous embolism.

**Clinical discussion:**

Fibrocartilaginous embolism is a rare cause of spine infarction. There is still little understanding of the underlying cause of FCE. Most cases occur sporadically in people without a family history of the disease, such as the authors’ case, and diagnosis is based on imaging of the spinal cord and ruling out other causes of a blockage in the vascular system within the spinal cord, infectious and inflammatory causes.

**Conclusion:**

When a practitioner suspects that a patient may have fibrocartilaginous embolism (FCE), they should take the patient’s history and do a neurological examination. An MRI is required since it is thought to be the most accurate method of diagnosing FCE.

## Introduction

Highlights

- Fibrocartilaginous embolism is a rare cause of spine infarction.
- There have only been 67 cases of fibrocartilaginous embolization of the spinal cord infarct reported in humans.
- A 29 female patient presented to our emergency department after a history of falling down.
- MRI findings point towards fibrocartilaginous embolism.

A fibrocartilaginous embolization (FCE) of the spinal cord is a rare condition caused by the migration of the fibrocartilaginous nucleus pulposus due to rupture of the intervertebral disc and the entry of a small piece of it into the spinal or vertebral blood vessel^1^, leading to spinal cord infarction due to blockage and obstruction of arterial branches supplying the spinal cord parenchyma. According to reports, FCE accounts for 5.5% of spinal cord infarctions^2^. The age of onset is bimodal, with a peak in adolescents and a second peak in older adults^3^.

The symptoms of FCE often develop after a history of minor trauma, vigorous exercise, or even triggering incidents that go unnoticed, such as lifting, straining, or falling, which can lead to rupture of the intervertebral disc^2^. The clinical signs appear as a result of altered blood flow to the spinal cord, which causes a lack of oxygen to the neurons, which can become dysfunctional and lead to the neurological signs associated with FCE, which may vary from weakness to complete paralysis.

This case study shows a rare case of fibrocartilaginous embolism in a young female patient presented to our emergency department after a history of falling down, providing valuable insights into neurological development and highlighting the diagnostic challenges associated with such cases. This work has been reported in line with the SCARE 2023 criteria^4^.

### Case presentation

A 29-year-old female patient presented to the emergency department after a history of falling down. The patient was in her usual state of health when she slipped on a wet floor at work 2 weeks prior to admission. She didn’t develop any symptoms immediately after the event; she continued her work and arrived home without any complaints. On the morning of the second day after falling, the patient started complaining of general weakness, malaise, and dizziness. Then she felt weakness and an inability to move her right hand.

Symptoms worsened over the next few hours as she lay at home and tried to sleep. By the afternoon, when she woke up, she couldn’t get out of bed. After she returned to consciousness, she felt bilateral upper and lower limb weakness and numbness. The condition started with left lower limb pain and weakness, gradually increasing and then shifting to the right lower limb, then ascending to her abdomen and chest, and then to her distal upper limbs.

The next day, the patient sought medical advice at our hospital. On examination, the patient’s vital signs were normal; she was immobile in bed, conscious, oriented, and alert to place, time, and person with a normal resting heart rate and no murmur. The abdomen was soft and lax. The patient has intact sensation all over the body with hyperaesthesia. The muscle power of all limbs was 0/5, and reflexes were absent. There’s no skin rash or lower limb oedema.

Laboratory investigation showed no evidence of infectious, autoimmune, inflammatory, or neoplastic causes (Table 1). A brain MRI revealed normal brain parenchyma and symmetrical cortical sulci in the absence of any indication of a space-occupying lesion. A long segment of abnormally high T2 signal was detected on a spinal MRI, measuring up to 8 cm in length. The segment involved the spinal cord, at least from (C3 to D1), and had a whole central grey matter of the cord involvement in the cross-section. This was linked to a high diffusion-weighted imaging (DWI) and low apparent diffusion coefficient (ADC) signal, which is consistent with cord ischaemia. There are no obvious enhancement components. To corroborate the diagnosis, an MRI scan of the cervical spine with contrast was also performed.

**Table 1:** The patient had mild anaemia, laboratory investigation demonstrated normal kidney function tests, lever function tests, and normal electrolytes.

	Test	Result	Normal range
Special tests	Random blood glucose	97	less than 140 mg/dl
Lumbar puncture (LP)	Colour	Straw	
	Turbidity	Turbid	
	Leucocyte	20	Less than 5 cells
	Erythrocyte	5000	0–10 cells
	Glucose	65	2\3rds serum glucose
	Protein	33	23–38
Haematology	WBC	5.5	(5.0–10.0)×10^3^
	RBC	4.72	4.2–5.4
	НЬ%	10.4	12–16
	HCT	31.9	37–48
	MCV	67.6	82–92
	MCH	22.1	27–31

Lumber puncture was done to rule out other causes such as acute inflammatory myelopathy and Guillain–Barre syndrome. Spinal fluid analysis is generally unrevealing except for elevation of leucocyte and erythrocyte elevation (may be due to traumatic LP). There’s no elevation in protein levels and no pleocytosis.

Hb, haemoglobin; HCT, haematocrit; MCH, mean corpuscular hemoglobin; MCV, Mean corpuscular volume; RBC, red blood cell; WBC, white blood cell.

**Figure 1:**
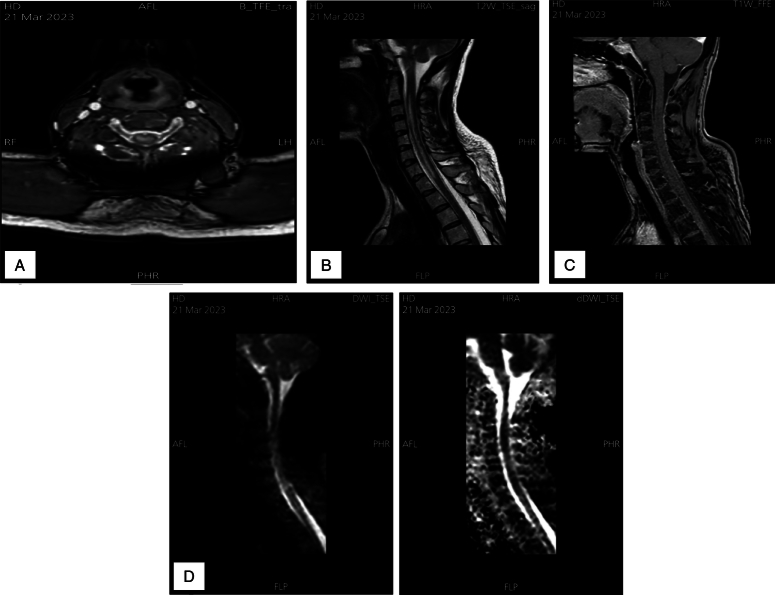
(A) Axial T1 wighted image (B) sagittal T2 image; show abnormally hyper-intense T2 signal (C) sagittal T1 wighted image post-contrast shows no abnormal enhancement. (D) High diffusion-weighted imaging signal intensities involving the centre of the spinal cord from C3 down to D1, with corresponding low signal of apparent diffusion coefficient image represent true diffusion restriction.

The majority of the transverse spinal cord had a hyper-intense T2 signal between C2 and T1, which suggests a significant spinal cord infarction or inflammation. Furthermore, the signal is weaker at the C4–C5 and C5–C6 intervertebral discs. And there was no T1 post-contrast abnormal enhancement. These findings may point towards possible fibrocartilaginous embolism (Figure 1).

Next, a brain and neck computed tomography angiography (CTA) was performed. It proved that both the vertebral and common carotid arteries (CCA) originated normally. No filling defect or atherosclerotic alterations, normal bilateral internal and external carotid arteries, and normal bifurcation of both CCAs. It was concluded that there is no indication of significant vascular damage because no aneurysmal changes were observed. This verified the fibrocartilaginous embolism diagnosis.

During hospitalization the patient received pulse steroids (Solumedrol) for 3 days, the patient’s sensation improved, and she became conscious and oriented with quadriplegia and urinary incontinence. Later on, for the last eight months, the patient has undergone physical therapy. She continues to receive physiotherapy sessions to enhance her quality of life, and she can now walk with the assistance of crutches.

## Discussion

FCE is an uncommon and under-recognized cause of ischaemic myelopathy of the spinal cord. There have only been 67 cases of FCE-related spinal cord infarct reported in humans with a female predominance^5^. Embolization may be either purely arterial 50%, or arterial and venous 50%^6^.

Decreased blood flow due to vascular injury, compression, hypotension, other impairment, or thromboembolism to spinal arteries can cause Ischaemic spinal cord infarction^7^. Several mechanisms have been proposed to explain FCE-spinal cord-related infarcts. The most common is the migration of nucleus pulposus material originating from Schmorl’s nodes following acute disk herniation to the vasculature, resulting in embolization into spinal cord vessels. As a result, FCE is often misdiagnosed as transverse myelitis at the beginning^8^.

Frequently, FCE may be misdiagnosed as acute inflammatory myelopathy, as acute spinal shock with flaccid paralysis may be present in both of them^1^. Regardless of the underlying cause, cerebellar spinal fluid (CSF) analysis in spinal cord infarction typically reveals increased protein but can also be normal (such as in our case). Also, FCE, In contrast to inflammatory cord lesions, it does not exhibit elevated IgG index or pleocytosis^9^. An MRI of the spinal cord in an infarction usually reveals T2 hyper-intense lesions in a vascular distribution. These lesions, in contrast to those of an inflammatory cord lesion, usually do not enhance with gadolinium and can appear 12–48 h after the beginning of symptoms^10^.

Other differential diagnoses of FCE include Guillain–Barre syndrome (GBS). In our case, GBS was ruled out because lumber puncture was done and CSF analysis demonstrated normal protein and elevation in leucocyte count. In contrast, during the acute phase of GBS, characteristic findings on CSF analysis include albumin-cytologic dissociation, which is an elevation in CSF protein (>0.55 g/l) without an elevation in white blood cells, so according to the Spine MRI Findings and the clinical picture of the patient, the appropriate diagnosis was FCE^2^.

Currently, FCE is diagnosed based on clinical grounds and is only verified through a biopsy for histopathologic analysis, typically at an autopsy. A schematic approach is used to diagnose FCE in five steps^2^.

Step 1: Establish the clinical syndrome of myelopathy, the sensory level being most useful^8^. Step 2: Exclude traumatic and compressive etiologies of myelopathy by history and imaging using spine CT or MRI with and without contrast. Step 3: Exclude inflammatory etiologies of myelopathyhttps, mainly by^10^: Absence of pleocytosis or increased IgG index in CSF, and Absence of gadolinium enhancement on MRI of the spine. Step 4: Establish the diagnosis of spinal cord infarction. This requires the above (Steps 1–3) plus one “Major” criterion or two “Minor” Criteria.

Major criteria:  Clear vascular distribution by exam, as sparing of proprioception or vibratory sensation^11^, Clear vascular distribution on imaging modalities, mainly axial views of MRI of the spine^12^.  Radiologic changes, mainly MRI T2 hyper-intensity, in the vertebral body or intervertebral disc adjacent to the infarction^13^. Minor criteria: Accompanying new onset neck or back pain^13^. Symptom progression to nadir or near nadir in less than 4–8 h^11^. Initial unremarkable MRI of the spinal cord with subsequent evolution of an intra-parenchymal lesion^12^.

Step 5: Establish the high likelihood of FCE. This requires the absence of other more common etiologies of spinal cord infarction, mainly aortic pathologies. So, according to these criteria, our patient was diagnosed as a case of FCE because she has all the above (Steps 1–3) plus one “major” criterion, which is MRI T2 hyper-intensity. In our case, MRI findings demonstrated long segments of non-expanded, non-enhancing cervical spine infarction, at least from C3 to D1, raising the suspicion of ischaemia due to a cut in blood supply. This occurs when materials that are usually found within the vertebral disc of the spine enter into the nearby vascular system (veins and arteries) and block one of the spinal cord vessels. In our patient, the clinical history, cerebrospinal fluid, and MRI features all pointed towards FCE^9^.

The clinical picture depends on the location of the immobilization but overall includes progressive myelopathy, weakness and severe back pain. Usually, myelopathy is predominant in the cervical (69%) and lumbosacral (22%) segments^9^. Our case developed symptoms on the next day after the trauma, although acute symptoms typically occur within minutes to hours, FCE has been reported in patients with symptom-free intervals of days following the presumed precipitating event. Similarly, FCE has occurred in patients who suffered seemingly minor or no trauma^14^.

Fibrocartilaginous embolism is a rare cause of spine infarction. There is still little understanding of the underlying cause of FCE. Most cases occur sporadically in people without a family history of the disease, such as our case, and diagnosis is based on imaging of the spinal cord and ruling out other causes of a blockage in the vascular system within the spinal cord, infectious and inflammatory causes^2^.

Prognosis is favourable in FCE, and it is largely dependent on the degree of spinal cord injury. (54% of patients with spinal cord infarction show improvement)^7^. Also, our patient improved and started walking after eight months of physiotherapy.

The main goals of treatment in FCE are to improve quality of life and prevent further complications through the use of pharmacologic and physical therapy. The Patients have been given oral and intravenous steroids, but there has been no discernible benefit. Future studies might focus on creating intravenous fibrinolytic or chondrolytic therapy to dissolve the FCE. This can be given if there is a clinical suspicion of FCE in the acute setting in an effort to protect spinal cord integrity before the ischaemic injury occurs.

## Conclusions

Spinal cord infarction is uncommon, and this case of fibrocartilaginous embolism demonstrates this point. Because FCE is totally curable and readily relieved, this disorder has clinical significance. Therefore, this FCE case highlights the difficulties in diagnosing and treating this uncommon neurological condition. When a practitioner suspects that a patient may have this illness, they must develop differential diagnoses based on the patient’s condition using a thorough approach that includes taking the patient’s history and doing a neurological examination. An MRI is required since it is thought to be the most accurate method of diagnosing FCE.

## Ethical approval

Not applicable.

## Informed consent

Written informed consent was obtained from the patient for publication of this case report and accompanying images. A copy of the written consent is available for review by the Editor-in-Chief of this journal on request.

## Consent for publication

All authors provide consent for publication.

## Source of funding

There are no funding sources.

## Author contribution

All authors fulfil the authorship criteria because of their substantial contributions to the conception, design, analysis, and interpretation of the data. The work’s conception and design: all authors. Paper writing, and article revision: all authors. Final revision and approval: all authors.

## Conflicts of interest disclosure

The authors declare that they have no competing interests.

## Research registration unique identifying number (UIN)

There are no funding sources.

## Guarantor

Afnan W. M. Jobran.

## Data availability statement

Not applicable.

## Provenance and peer review

Not commissioned, externally peer-reviewed.
